# Effect of bovine bone collagen oligopeptides on wound healing in mice

**DOI:** 10.18632/aging.202750

**Published:** 2021-03-10

**Authors:** Di Li, Jin-wei Ren, Teng Xu, Lin Li, Peng Liu, Yong Li

**Affiliations:** 1 Department of Clinical Nutrition, Peking University People’s Hospital, Beijing 100044, China; 2 Department of Nutrition and Food Hygiene, School of Public Health, Peking University, Beijing 100191, China

**Keywords:** bovine bone, collagen oligopeptides, wound healing

## Abstract

Impaired wound healing often brings a set of problems in clinical practice. This study aimed to observe the wound healing potential of bovine bone collagen oligopeptides (BCOP) in mice. After an operation, mice in BCOP-treated groups were given intragastric administration of BCOP, while others were administered vehicle. Mice were sacrificed at different points. The wound healing condition and the tensile strength were observed, serum biochemical indexes and mRNA expression of level of related genes were measured. Compared with the normal control group, albumin (ALB), prealbumin (PA), transferrin (TRF), hydroxyproline (Hyp) levels and tension strength in the BCOP-treated groups increased significantly (*p* < 0.05). A pathological report showed that neutrophil granulocyte in the BCOP-treated groups decreased, while blood capillary and fibroblasts increased. The levels of serum inflammation indexes like interleukin (IL)-8, tumor necrosis factor (TNF)-α, chemokine (C-C motif) ligand 2 (CCL2) and C-reactive protein (CRP) significantly decreased in full-thickness incision model, whereas increased in full-thickness excision model (*p* < 0.05). Furthermore, IL-10, stromal cell-derived factor-1 alpha (SDF-1α) levels and the mRNA expression of vascular endothelial growth factor (VEGF) significantly increased in both models (*p* < 0.05). These results suggested that oral administration of BCOP could promote wound healing in mice.

## INTRODUCTION

Poor wound healing not only causes physical pain and inconvenience to patients, but also brings a series of psychological problems, such as anxiety, inferiority, and isolation. In addition, hard-to-heal wounds may lead to further deterioration in the quality of life of patients and bring a heavy economic burden [1–2]. Thus, the development of a reliable and efficacious therapy in the management of wound healing remains a crucial clinical problem. However, current therapeutic methods in the management of wounds have many limitations [3–4]. As the outer barrier, the skin is frequently challenged by various external damage. In order to enhance the regeneration capacity of endogenous tissues, a great deal of research has been envisaged to develop better healing agents.

Nutrition intervention has become an effective and widely used method to enhance the immune response, reduce the degree of inflammatory response and fight diseases. To date, a variety of bioactive peptides have been isolated from animals, plants and microorganisms. In addition to being easily absorbed and digested, they also have a wide range of physiological functions, including collagen synthesis-improvement, antimicrobial, antioxidant or cholesterol-lowering or immunomodulatory activities [5–6]. Moreover, some peptides are capable of performing multiple physiological functions simultaneously [7]. Accordingly, plenty of food-derived bioactive peptides are drawing more and more attention in the fields of treatment to promote wound healing after surgery.

Bone is a special connective tissue, which is mainly composed of bone matrix. The bone matrix consists of embedded adhesion of hydroxyapatite collagen. Ninety percent of the protein in bovine bone is fibrous collagen. Moreover, the fibrous collagen is one of the most abundant proteins in the body, which causes it to become a potential candidate for clinical nutrition. Meanwhile, they may exert many beneficial biological activities like anti-aging, anti-inflammatory, antioxidant, immunological enhancement, as well as wound healing activities [8]. As a short chain polypeptides derived from bovine bone, bovine bone collagen oligopeptides, (BCOP) are easily absorbable nitrogen source, furthermore, they have variety of pharmacological activities, including immunomodulatory, antioxidant, anti-aging, anti-inflammatory, angiogenesis-promoting activity and so on [9]. Our former studies showed that oligopeptides powder of marine fish collagen had a benefit effect on wound healing, yet studies between BCOP and wound healing is unavailable [10–11].

Round wounds and incision wounds are two common types of wounds in clinical practice. At present, most animal models for wound healing experiments only use the full-thickness excision wound model [12–14]. In this study, not only the full-thickness excision wound model was adopted, but also the full-thickness incision wound model deep into the abdominal cavity was pioneered by our research team [15]. This model can simulate the whole process of clinical surgery and more comprehensively reflect the dynamic process of the patient's body changes after surgery. In addition, the combination of these two models can better reflect the complexity and diversity of wounds in clinical practice.

Therefore, in this study, we used two wound models to investigate whether BCOP can improve wound healing in mice and its possible mechanisms. To this end, we intragastrically administered deionized water as vehicle, and BCOP to mice and evaluated the effects of BCOP on wound healing compared with vehicle.

## RESULTS

### General information, total protein and albumin concentrations in serum

Despite weight loss, no differences were found between the vehicle and the BCOP-treated groups during the experiment.

The levels of ALB, PA and TRF were presented in Table 1. Compared with NC, ALB, PA and TRF levels were significantly reduced in the BCOP-treated groups on day 7 and 11 in full-thickness incision model, and on day 8, 12 and 16 in full-thickness excision model (*p* < 0.05).

**Table 1.** Serum ALB, PA and TRF concentration in full-thickness incision and excision wound model from vehicle- and BCOP-treated mice.

	**Incision wound model**				**Excision wound model**		
	**DAY 3**	**DAY 7**	**DAY 11**	**DAY 4**	**DAY 8**	**DAY 12**	**DAY 16**
The level of ALB (μg/L)							
NC	268.60±27.29	276.17±42.48	288.97±48.42	265.61±34.96	214.93±34.25	213.27±39.30	228.67±33.43
TabBCOP-L	385.63±35.79^*^	442.13±53.01^*^	323.46±35.59	351.73±19.34^*^	374.42±36.49^*^	344.28±38.09^*^	241.44±27.07
BCOP-M	301.01±60.44	537.56±37.23^*^	449.74±46.00^*^	372.35±21.89^*^	373.90±30.95^*^	445.32±55.51^*^	312.05±28.95^*^
BCOP-H	403.04±19.33^*^	475.18±57.11^*^	542.60±53.42^*^	480.03±31.92^*^	497.96±42.55^*^	339.88±30.74^*^	323.29±35.42^*^
The level of PA (μg/mL)							
NC	31.62±2.75	35.17±4.04	20.61±3.87	17.95±3.17	16.47±3.19	22.48±3.95	23.71±4.41
BCOP-L	47.29±3.34^*^	41.01±3.26^*^	28.67±4.09	26.80±2.30^*^	30.50±3.17^*^	27.67±3.05^*^	33.83±4.19^*^
BCOP-M	39.71±3.17^*^	40.71±4.21^*^	39.98±3.32^*^	33.46±2.19^*^	27.03±3.40^*^	31.08±3.51^*^	39.03±3.42^*^
BCOP-H	36.68±3.13^*^	42.13±4.29^*^	47.84±3.89^*^	38.25±2.88^*^	34.94±3.15^*^	50.15±3.94^*^	35.74±4.68^*^
The level of TRF (nmol/L)							
NC	112.75±26.62	196.65±28.43	111.87±19.38	100.40±16.74	114.71±15.64	106.91±14.22	92.19±12.34
BCOP-L	199.37±27.56^*^	214.82±21.69^*^	246.14±21.89^*^	133.85±18.92^*^	155.43±13.22^*^	160.12±14.96^*^	138.96±12.77^*^
BCOP-M	198.26±16.48^*^	228.05±25.99^*^	261.86±23.17^*^	214.02±21.89^*^	147.08±11.59^*^	170.59±19.14^*^	152.81±14.92^*^
BCOP-H	223.34±27.07^*^	184.71±21.06^*^	216.47±22.15^*^	190.44±18.03^*^	220.25±18.38^*^	212.92±14.67^*^	207.79±16.09^*^

Values were presented as mean ± SD. ^*^Compared with the NC, statistical significance was set at *p* < 0.05. NC, normal control group normal control group; BCOP-L, 0.75 g/kg bovine bone collagen oligopeptides group; BCOP-M, 1.50 g/kg bovine bone collagen oligopeptides group; BCOP-H, 3.00 g/kg bovine bone collagen oligopeptides group.

### Wound closure and tensile strength

**Figure 1:**
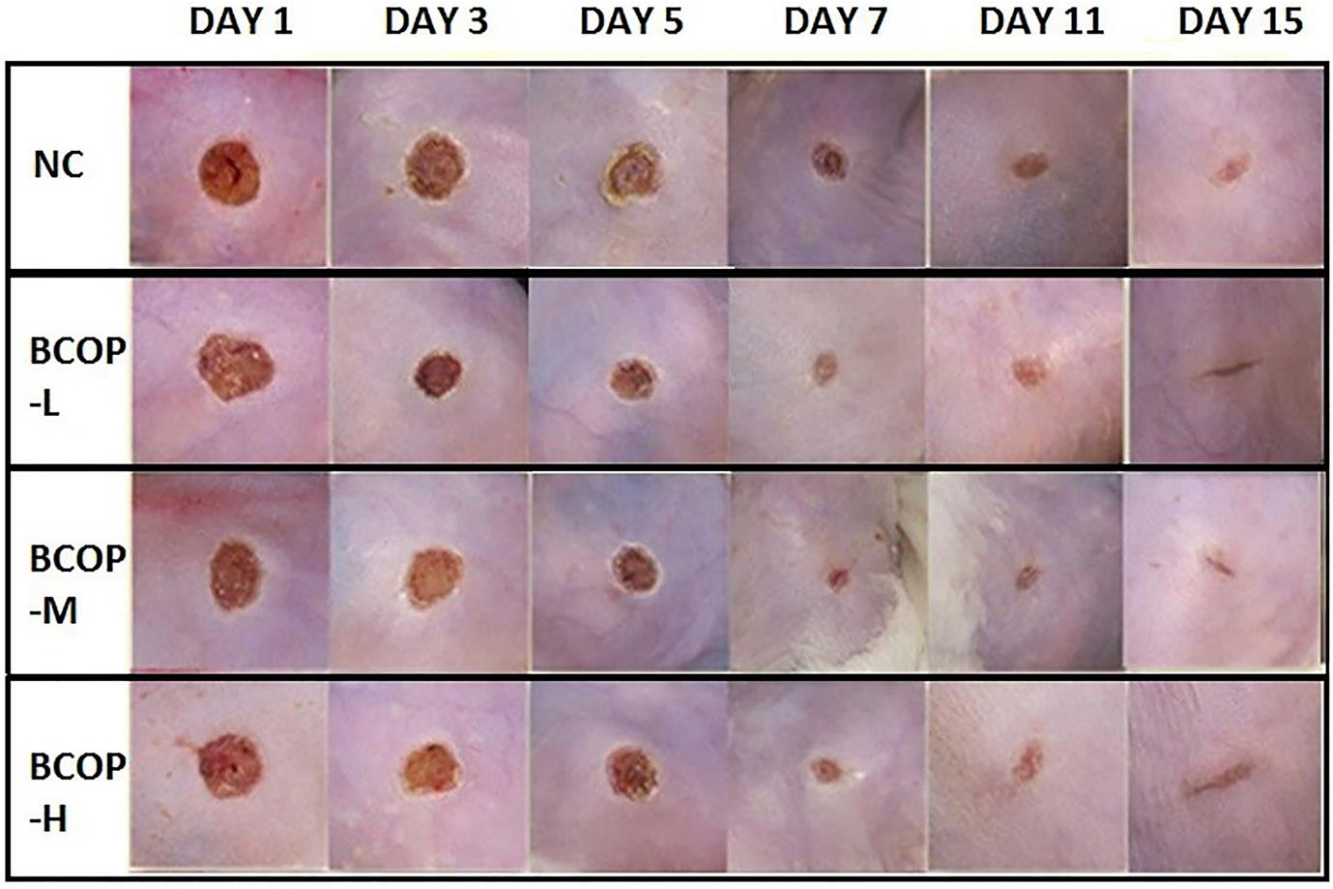
**Effect of BCOP on macroscopic images of wounds in mice.** Representative photos of wounds in mice treated with either vehicle- or BCOP on day 1, 3, 5, 7, 11 and 15. NC, normal control group; BCOP-L, 0.75 g/kg bovine bone collagen oligopeptides group; BCOP-M, 1.50 g/kg bovine bone collagen oligopeptides group; BCOP-H, 3.00 g/kg bovine bone collagen oligopeptides group.

**Figure 2:**
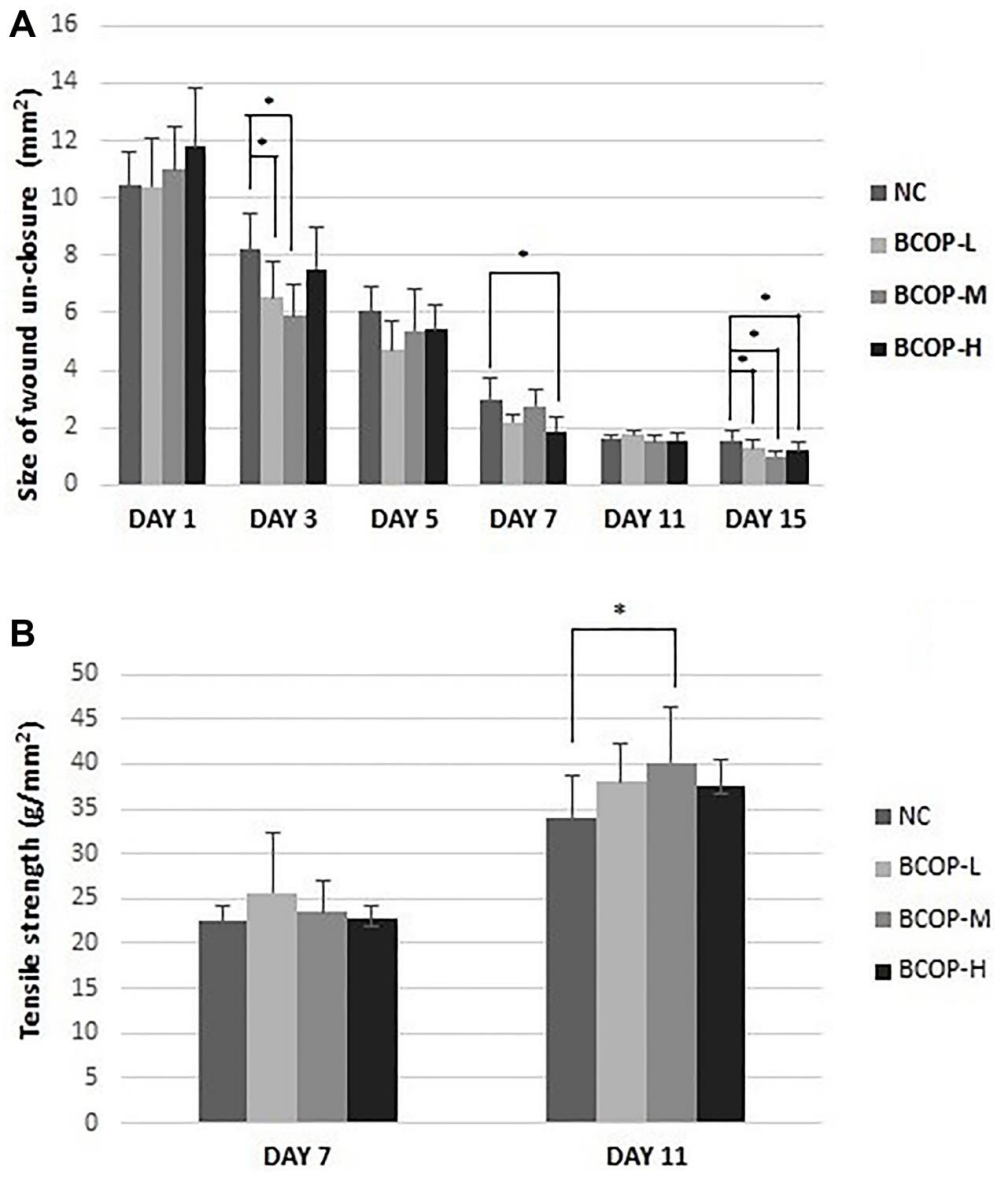
**The size of wound un-closure.** (**A**) in excision wound tissue and the tensile of strength (**B**) in incision wound tissue of mice treated with vehicle and BCOP. NC, normal control group; BCOP-L, 0.75 g/kg bovine bone collagen oligopeptides group; BCOP-M, 1.50 g/kg bovine bone collagen oligopeptides group; BCOP-H, 3.00 g/kg bovine bone collagen oligopeptides group. Values were presented as mean ± SD. ^*^Compared with NC, statistical significance was set at *p* < 0.05.

We first studied the effect of BCOP on wound closure in the excision wound model, and compared the results with their vehicle controls (Figure 1). As illustrated in Figure 2A, the size of un-closure in the groups treated with BCOP was 6.54±1.26 mm^2^ (BCOP-L), 5.89±1.14 mm^2^ (BCOP-M) and 7.55±1.42 mm^2^ (BCOP-H) on day 3, 2.21±0.27 mm^2^ (BCOP-L), 2.74±0.57 mm^2^ (BCOP-M) and 2.99±0.71 mm^2^ (BCOP-H) on day 7, and 1.27±0.35 mm^2^ (BCOP-L), 1.02±0.16 mm^2^ (BCOP-M) and 1.19±0.34 mm^2^ (BCOP-H) on day 15, respectively. While in NC, it was 8.20±1.28 mm^2^, 2.99±0.71 mm^2^ and 1.56±0.33 mm^2^, respectively. Compared with NC, wounds treated with BCOP were smaller in the size of un-closure (*p* < 0.05). The time required for complete epithelialization of the excision wound is an important parameter to assess the wound healing process. It was found that in BCOP-treated mice, epithelialization was completed on day 7, while in vehicle-treated animals, the completed epithelialization of the excision wound was extended to day 11.

At the beginning, the wound had almost no breaking strength because only the clot would hold the edges together. Wound tensile strength measurements on day 7 and 11 indicated that lower wound tensile strengths were measured in vehicle-treated mice. Compared with vehicle-treated mice, BCOP-treated mice had higher wound tensile strength on day 11 (*p* < 0.05). The differences among groups were statistically significant (Figure 2B).

### Histological analysis

**Figure 3:**
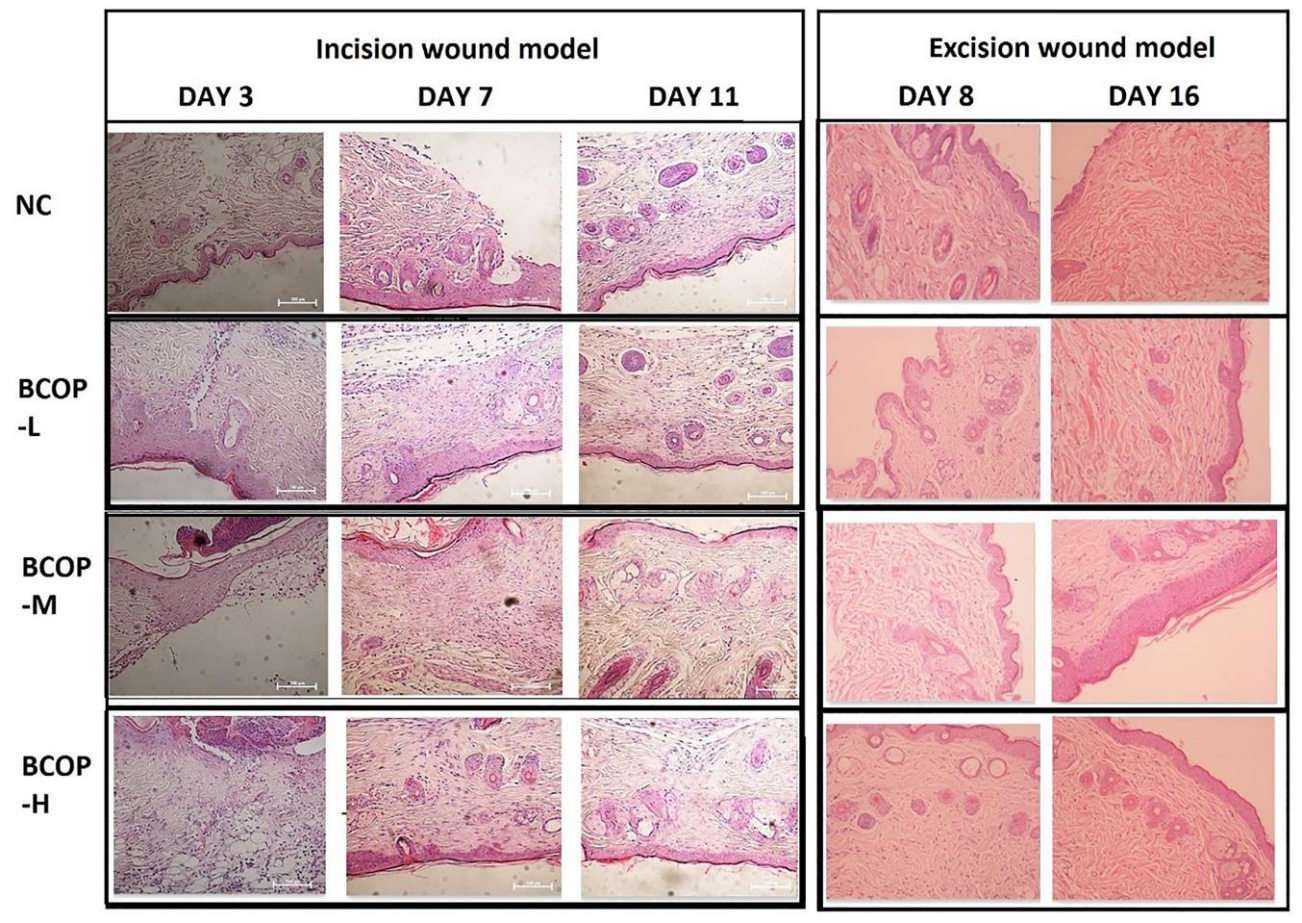
**Effect of BCOP on wounds histology (HE staining) in mice.** The incision site of NC showed irregular arrangement of collagen bundles, loose accumulation, and only moderate fibroblasts in the wound. BCOP-treated wound sites showed dense and compact collagen bundles with more fibroblasts, increased angiogenesis, and continuous epithelialization, reflecting faster wound healing compared to vehicle-treated groups. Magnification, × 200; NC, normal control group; BCOP-L, 0.75 g/kg bovine bone collagen oligopeptides group; BCOP-M, 1.50 g/kg bovine bone collagen oligopeptides group; BCOP-H, 3.00 g/kg bovine bone collagen oligopeptides group.

Histological assessment, including the extent of neutrophil and fibroblast infiltration, collagen deposition, angiogenesis and epithelialization in the wound area, was shown in Figure 3. Compared to NC, mice in the BCOP-treated groups showed enhanced fibroblast infiltration, increased blood vessel formation, collagen deposition, and epithelialization. In groups treated with BCOP, the number of infiltrating fibroblasts in the subcutaneous tissue increased significantly on day 7 (full-thickness incision model) and day 8 (full-thickness excision model). In addition, on day 11 (full-thickness incision model) and day 16 (full-thickness excision model), a large amount of collagen deposition was observed in BCOP-treated groups. Moreover, on day 11 (full-thickness incision model) and day 16 (full-thickness incision model), the epithelialization of BCOP-treated groups was significantly larger than that of the NC.

### Serum inflammatory response

The indicators of inflammatory response were measured, and the results were shown in Table 2, 3 and 4. The levels of CRP, IL-8, IL-10, TNF-α and CCL2 in serum were measured as indicators of inflammatory activity. In full-thickness incision model, after the intervention, the serum inflammation indexes including CRP, IL-8, TNF-α, and CCL2 significantly decreased (*p* < 0.05); while IL-10 levels significantly increased (*p* < 0.05). However, in full-thickness excision model, all the inflammation indexes including IL-8, IL-10, TNF-α, CCL2 and CRP significantly increased (*p* < 0.05).

**Table 2.** Serum CRP concentration in incision and excision wound model from vehicle- and BCOP-treated mice.

**Group**	**Incision wound model**				**Excision wound model**		
	**DAY 3**	**DAY 7**	**DAY 11**	**DAY 4**	**DAY 8**	**DAY 12**	**DAY 16**
NC	0.50±0.20	0.25±0.10	0.38±0.21	0.40±0.19	2.21±0.46	0.41±0.25	0.20±0.12
BCOP-L	0.42±0.24	0.56±0.28^*^	0.29±0.24	1.15±0.69^*^	1.81±0.54	0.63±0.23	0.25±0.16
BCOP-M	0.32±0.13	0.50±0.29^*^	0.35±0.19	1.18±0.87^*^	1.70±0.37	0.64±0.26	0.55±0.41^*^
BCOP-H	0.42±0.38	0.36±0.23	0.17±0.09^*^	0.69±0.36	1.55±0.29^*^	0.69±0.21^*^	0.20±0.13

Values were presented as mean ± SD. ^*^Compared with the NC, statistical significance was set at *p* < 0.05. NC, normal control group; BCOP-L, 0.75 g/kg bovine bone collagen oligopeptides group; BCOP-M, 1.50 g/kg bovine bone collagen oligopeptides group; BCOP-H, 3.00 g/kg bovine bone collagen oligopeptides group.

**Table 3.** Serum inflammatory response in incision wound model mice treated with vehicle and BCOP on day 3, 7 and 11.

	**DAY 3**		**DAY 7**		**DAY 11**	
	**Mean**	**SD**	**Mean**	**SD**	**Mean**	**SD**
The level of IL-8 (pg/L)						
NC	102.91	9.09	110.29	7.86	114.61	7.54
BCOP-L	79.24	10.19	112.01	9.87	81.77^*^	9.29
BCOP-M	90.9	9.57	84.41^*^	11.42	93.27^*^	10.12
BCOP-H	86.26	14.9	58.01^*^	8.98	80.80^*^	10.42
The level of IL-10 (pg/mL)						
NC	508.07	70.39	533.71	86.23	499.31	75.09
BCOP-L	825.55^*^	114.94	710.37^*^	56.71	569.32^*^	73.9
BCOP-M	899.85	94.8	632.43^*^	85.35	743.78^*^	73.26
BCOP-H	625.79	62.26	454.28	77.69	700.89^*^	94.4
The level of TNF-α (ng/L)						
NC	498.29	46.91	625.99	69.02	611.05	65.08
BCOP-L	503.99	41.71	456.88^*^	59.89	365.92^*^	58.41
BCOP-M	461.23	79.06	518.22^*^	64.51	570.31	52.09
BCOP-H	590.36	71.59	594.22	97.76	318.58^*^	64.29
The level of CCL2 (pg/ml)						
NC	1525.89	144.69	1474.83	142.54	1723.02	180.14
BCOP-L	1150.51^*^	143.81	1057.99^*^	145.77	1018.70^*^	194.77
BCOP-M	775.37^*^	140.29	1044.19^*^	111.9	1219.20^*^	75.27
BCOP-H	1303.57	128.91	1287.58	130.32	1729.98	165.4
The level of SDF-1α (ng/L)						
NC	172.86	41.33	254.75	43.64	164.44	36.84
BCOP-L	265.44^*^	25.84	313.76^*^	34.55	265.02	37.99
BCOP-M	184.59	12.18	303.42^*^	36.69	294.16^*^	29.49
BCOP-H	234.22^*^	26.15	280.91	27.55	308.15^*^	37.19

Values were presented as mean ± SD. ^*^Compared with the NC, statistical significance was set at *p* < 0.05. NC, normal control group; BCOP-L, 0.75 g/kg bovine bone collagen oligopeptides group; BCOP-M, 1.50 g/kg bovine bone collagen oligopeptides group; BCOP-H, 3.00 g/kg bovine bone collagen oligopeptides group.

**Table 4.** Serum inflammatory response in excision wound model mice treated with vehicle and BCOP on day 4, 8, 12 and 16.

	**DAY 4**		**DAY 8**		**DAY 12**		**DAY 16**	
	**Mean**	**SD**	**Mean**	**SD**	**Mean**	**SD**	**Mean**	**SD**
The level of IL-8 (pg/L)								
NC	50.16	7.88	47.42	6.39	59.77	9.44	47.56	8.49
BCOP-L	74.86^*^	10.9	86.07^*^	4.70	79.16^*^	6.66	65.68^*^	11.75
BCOP-M	101.16^*^	6.82	82.42^*^	8.05	87.14^*^	8.6	108.76^*^	8.86
BCOP-H	89.01^*^	8.67	112.48^*^	8.60	93.30^*^	11.24	92.69^*^	7.00
The level of IL-10 (pg/mL)								
NC	467.06	58.82	413.71	72.52	368.09	48.56	395.54	50.09
BCOP-L	734.99^*^	48.56	569.36^*^	32.72	514.99^*^	61.87	488.09^*^	62.32
BCOP-M	761.16^*^	64.16	559.42^*^	70.00	560.72^*^	50.02	532.57^*^	38.78
BCOP-H	700.02^*^	61.46	711.89^*^	66.69	682.82^*^	48.51	742.23^*^	47.56
The level of TNF-α (ng/L)								
NC	369.64	60.6	342.92	47.18	278.77	39.76	320.62	56.7
BCOP-L	453.33^*^	56.6	425.05^*^	47.34	411.37^*^	24.24	389.01^*^	49.29
BCOP-M	563.44^*^	59.38	552.41	56.18	423.96^*^	41.19	519.25^*^	43.99
BCOP-H	675.38^*^	42.73	507.78^*^	34.44	639.03^*^	42.17	452.60^*^	40.75
The level of CCL2 (pg/ml)								
NC	984.14	86.45	889.24	92.75	867.29	127.84	932.04	135.03
BCOP-L	1175.68^*^	107.28	1327.49^*^	137.24	1244.69^*^	138.66	1016.32	145.88
BCOP-M	1724.06^*^	121.32	1570.65^*^	80.99	1454.34^*^	116.87	1228.95^*^	145.19
BCOP-H	1676.09^*^	78.78	1466.55^*^	69.96	1551.52^*^	110.85	1474.52^*^	120.02
The level of SDF-1α (ng/L)								
NC	155.72	22.37	176.29	19.89	178.02	23.59	142.22	21.71
BCOP-L	251.13^*^	23.73	253.29^*^	19.45	237.87^*^	22.73	217.01^*^	25.75
BCOP-M	259.40^*^	16.38	271.53^*^	23.25	312.73^*^	24.87	321.12^*^	27.93
BCOP-H	273.41^*^	21.49	327.74^*^	22.45	365.18^*^	30.21	354.57^*^	27.85

Values were presented as mean ± SD. * Compared with the NC, statistical significance was set at *p* < 0.05. NC, normal control group; BCOP-L, 0.75 g/kg bovine bone collagen oligopeptides group; BCOP-M, 1.50 g/kg bovine bone collagen oligopeptides group; BCOP-H, 3.00 g/kg bovine bone collagen oligopeptides group.

The level of SDF-1α in serum was measured as an indicator of vascular endothelial function. In comparison with NC, the levels of SDF-1α were significantly increased (*p* < 0.05) in BCOP-treated groups in both models.

### Quantitative analysis of hydroxyproline

**Figure 4:**
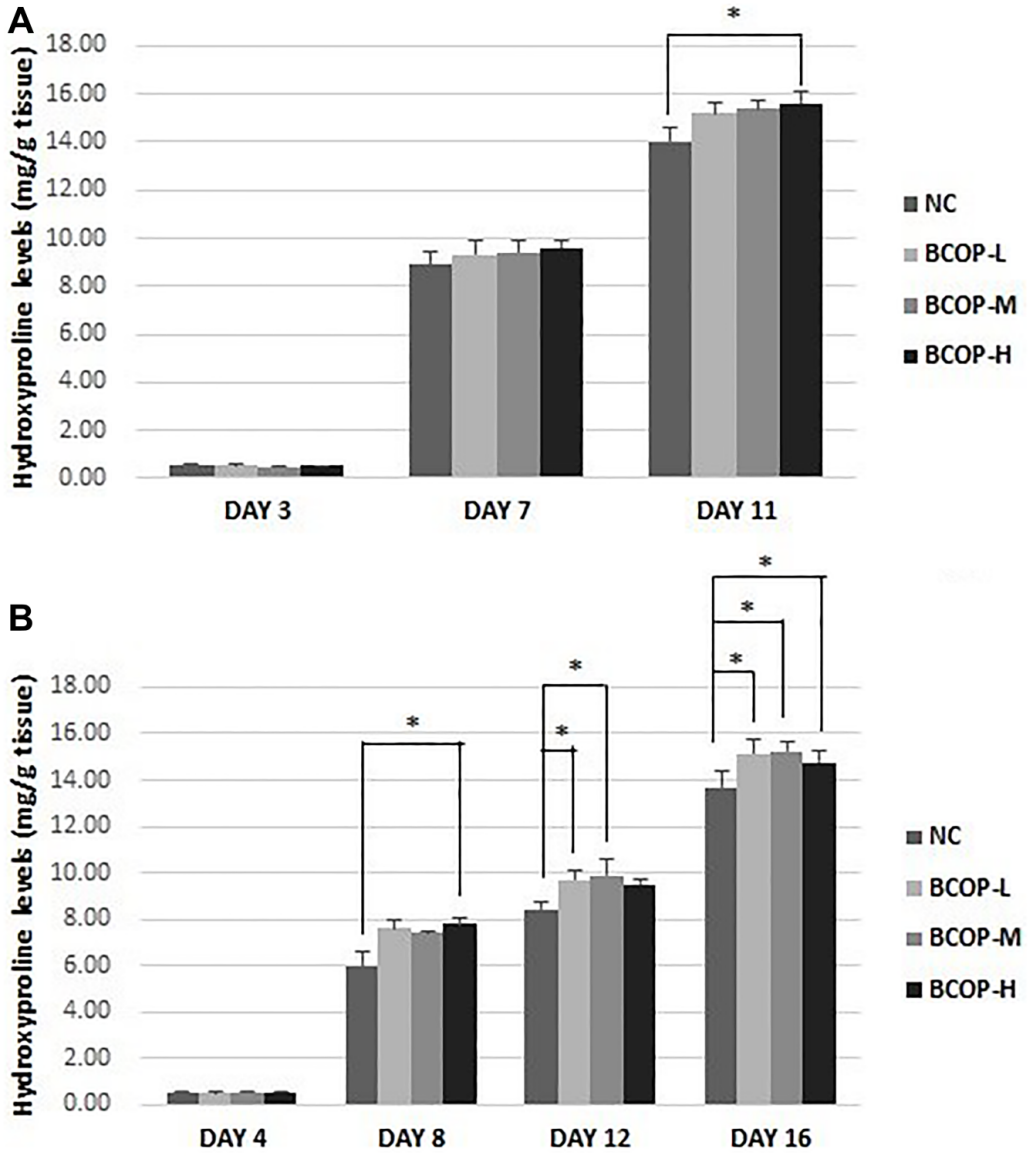
**Hydroxyproline levels in the full-thickness incision.** (**A**) and excision wound (**B**) tissue of mice treated with vehicle and BCOP. NC, normal control group; BCOP-L, 0.75 g/kg bovine bone collagen oligopeptides group; BCOP-M, 1.50 g/kg bovine bone collagen oligopeptides group; BCOP-H, 3.00 g/kg bovine bone collagen oligopeptides group. Values were presented as mean ± SD. ^*^Compared with the NC, statistical significance was set at *p* < 0.05.

Collagen deposition plays a key role in granulation tissue formation, and Hyp levels indicate collagen content. To confirm these histological observations, Hyp levels were measured in the lesion on day 3, 7 and 11 in full-thickness incision model and on day 4, 8, 12 and 16 in full-thickness excision model. The results showed, in the skin, compared with NC, the Hyp concentration increased significantly in the BCOP groups (on day 11 in full-thickness incision model and on day 8, 12 and 16 in full-thickness excision model) (*p* < 0.05) (shown in Figure 4).

### Analyses of VEGF mRNA

**Figure 5:**
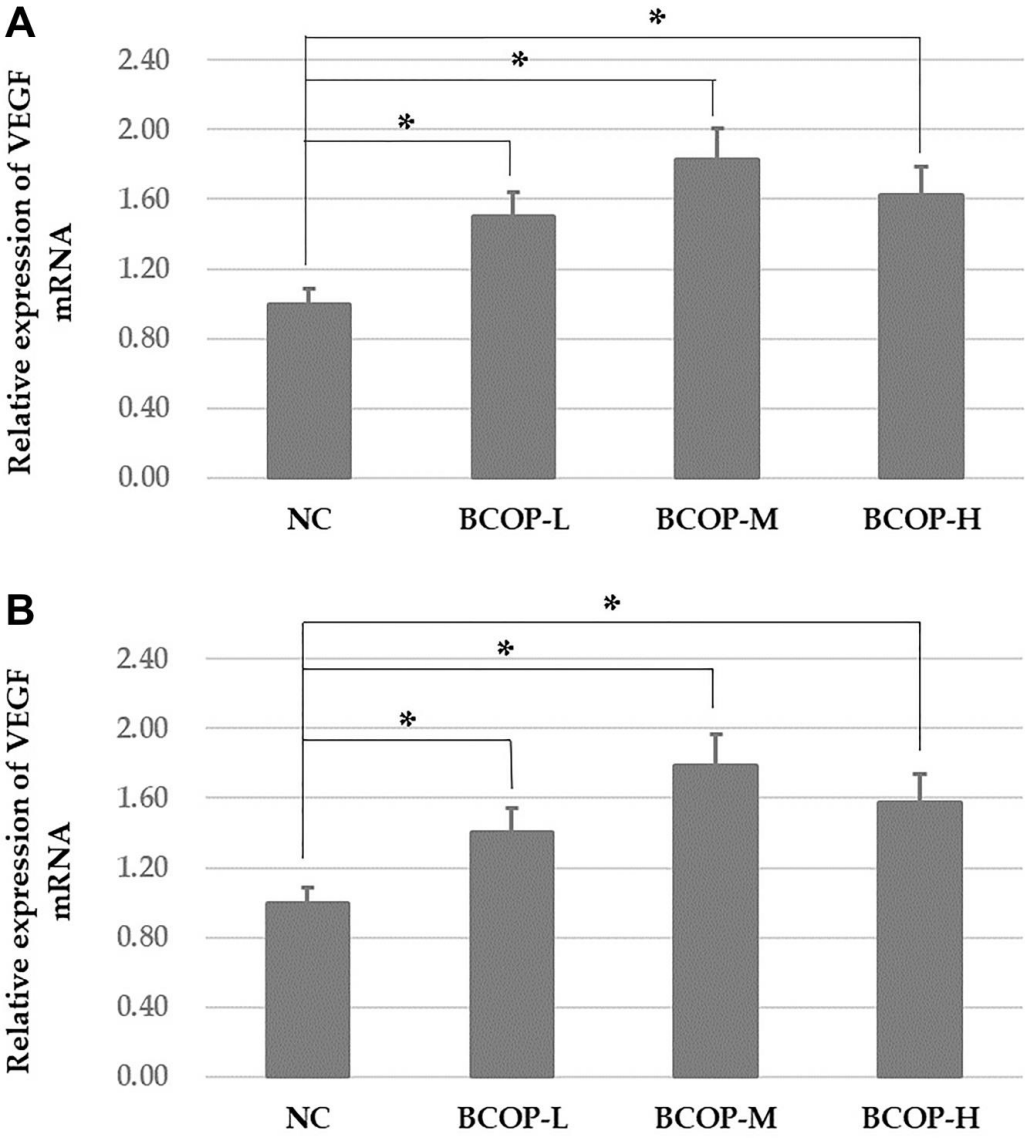
**VEGF mRNA levels in the full-thickness incision.** (**A**) and excision wound (**B**) tissue of mice treated with vehicle and BCOP. NC, normal control group; BCOP-L, 0.75 g/kg bovine bone collagen oligopeptides group; BCOP-M, 1.50 g/kg bovine bone collagen oligopeptides group; BCOP-H, 3.00 g/kg bovine bone collagen oligopeptides group. Values were presented as mean ± SD. ^*^Compared with the NC, statistical significance was set at *p* < 0.05.

As shown in Figure 5, on day 11 in full-thickness incision wound model and day 16 in full-thickness excision wound model, the mRNA expression of VEGF was both markedly increased in BCOP groups compared with NC (*p* < 0.05).

## DISCUSSION

Poor wound healing caused by trauma, surgery or chronic diseases affects millions of people worldwide every year. The cellular and molecular mechanisms of wound repair and regeneration are complex [16]. Wound healing is a complex process, usually artificially divided into three phases: inflammatory, proliferative and maturation [17]. In the present study, we found that BCOP derived from the hydrolysis of bovine collagen, whose main components are low molecular weight oligopeptides, have potential to promote wound healing. This study showed that oral administration of BCOP accelerated cutaneous wound healing in mice using two models.

Inflammation is a critical process in wound healing, and successful healing requires inflammation. In acute wound healing, the inflammatory response should occur rapidly and be sustained for 3–4 days to allow subsequent stages of wound healing to occur [18–20]. This requires inflammatory cells such as neutrophils and macrophages to migrate to the wound area and engulf necrotic tissue and microorganisms. Chronic wounds are still in a prolonged inflammatory state, which may be due to local and systemic defects [21]. To evaluate the inflammatory infiltrate, cytokines (such as IL-8, IL-10, TNF-α, CCL2 and CRP), indicative of the inflammatory activities in serum, were measured at different points (day 3, 7 and 11 in full-thickness incision model and day 4, 8, 12, 16 in full-thickness excision model). CRP, as an inflammatory biomarker, would rapidly increase during an acute inflammatory reaction, such as surgery. Interestingly, compared with the control, IL-8, TNF-α and CCL2 levels were elevated in full-thickness excision model, whereas they were reduced in full-thickness incision model. IL-8 and IL-10 can be pro-inflammatory or anti-inflammatory cytokines [22] but play vital roles in impaired angiogenesis, leukocyte recruitment and collagen deposition [23]. Because of that, the cytokines play as different roles between acute and chronic inflammations. Furthermore, IL-6 knockout mice show delayed wound healing [23], as well as reduced inflammatory response, epithelial regeneration, and granulation tissue formation [24]. SDF-1α induces endothelial cell migration and angiogenesis [25–26]. SDF-1α knockout mice show delayed epithelial regeneration, angiogenesis and collagen synthesis [27]. The significantly high expression of IL-8 and SDF-1α in mice of BCOP-treated groups indicates that there is an enhancement of healing through inflammatory cytokine expression. TNF-α expression has been detected in situations that inhibit and enhance [28–29] wound healing. Due to the expression of the other inflammatory cytokines in this work, the expression of TNF-α can be considered to indicate a good wound healing effect. Taken together, compared with NC, the administration of BCOP significantly modulated the inflammatory response and increased the number of fibroblasts in wound area. The positive effect of BCOP on the inflammatory response of the lesion may be explained by the NO synthesis stimulated by BCOP [30–31].

It was confirmed by a significant increase in the rate of wound closure, tensile strength and enhanced epithelialization by histological evaluation. Wound closure and tensile strength were related to a complex and well-planned interactions of cells, extracellular matrix and cytokines. The increased rate of wound closure and tensile strength in BCOP-treated wounds is probably due to increased proliferation and transformation of fibroblast cells into myofibroblasts. Histological results also showed that fibroblasts proliferated and re-epithelialized in mice treated with BCOP. Early re-epithelialization and wound closure in BCOP-treated mice may also be related to increased proliferation of keratinocytes and their migration to the wound surface [32–33]. Fibroblasts are responsible for the synthesis, deposition, and remodeling of the extracellular matrix. After implantation into the wound, fibroblasts begin synthesis of the extracellular matrix. Collagen is the main protein of the extracellular matrix and is the component that ultimately contributes to the strength of the wound [18]. A significant increase in closure, tensile strength, Hyp content and collagen levels were also observed in BCOP-treated wound tissues, which was further supported by histopathological studies and gain in granulation tissue angiogenesis. The enhanced level of Hyp in BCOP-treated mice probably contributes to strengthen the regenerated tissue. In the present study, the optimal dose of BCOP was 1.50 g/kg in mice, which meant it had dosage-dependent manners under a dosage range. There are currently few studies about BCOP in human. Further studies are needed to explore the optimal dose of BCOP to generate their promoting wound healing effects in humans.

Angiogenesis during wound repair has a dual function: it provides essential nutrients and oxygen to the wound site and promotes the formation of granulation tissue [19, 34–35]. In this study, the serum SDF-1α level of crucial factor that supports tissue regeneration [36]. In the process of wound healing, SDF-1α is responsible for mice treated with BCOP was significantly increased, providing evidence that BCOP can increase angiogenesis. In addition, histological evaluation showed increased angiogenesis in granulation tissue of mice treated with BCOP. It has been confirmed that SDF-1α plays an important role in lymphocyte homing, chemotaxis and secretion of angiogenic factors [37]. Additionally, SDF-1α has been demonstrated to be a recruiting mesenchymal stem cells and the release of their growth factors to injured tissues, besides, it can also increase wound healing rate and new blood vessel formation [38]. Immunohistochemical staining showed that SDF-1α could increase the expression of vascular endothelial growth factor (VEGF) in normal cartilage, especially in superficial regions [39]. VEGF provides a very important signal for angiogenesis and promotes revascularization in wound healing. VEGF improves angiogenesis during wound healing by stimulating the migration of endothelial cells through the extracellular matrix [40]. Consistent with our findings, Galiano *et al*. [34] demonstrated that local treatment with SDF-1α and VEGF in db/db mice could stimulate local angiogenesis to promote neovascularization at the wound site [36].

In addition, in order to eliminate confounding factors that dietary protein might affect wound healing, body weight and food consumption were measured daily. Compared with NC, no significant differences in protein intake or weight gain were found in BCOP-treated groups. However, compared to NC, ALB, PA and TRF levels were considerably increased during the whole experiment in the BCOP-treated groups. As malnutrition biomarkers, ALB, PA and TRF have been widely used for nutritional status evaluation after operation. Our study showed that BCOP increased the protein content of mice, and then improve their nutritional status.

In conclusion, this study suggested that BCOP might promote wound healing in mice after surgery. This may be due to its multi-target therapeutic properties, including reducing inflammatory response, improving angiogenesis and collagen deposition. Taken together, all evidence above makes it a new treatment direction for wounds in clinical practice. This natural food-derived nutritional formulation may be beneficial for wound healing. However, further research is needed to explore the mechanisms by which BCOP promotes wound healing, such as the PI3K/Akt /mTOR pathway.

## MATERIALS AND METHODS

### Materials and reagents

BCOP were derived from the bone of bovine by enzymatic hydrolysis and donated by Hua Peptide Bioengineering Co., Ltd. (Beijing, China). Oligopeptide samples were purified by high performance liquid chromatography (HPLC, Water Corp., Milford, M222KOA, USA) using a Phenomenex C18 column (10 mm × 250 mm), and the molecular weight distribution of the oligopeptide sample was determined by LDI-1700 matrix-assisted laser desorption ionisation time of-flight mass spectrometry (MALDI-TOF-MS) (Linear Scientific Inc., Reno, NV, USA). Then the amino acid composition was analysed with an automatic amino acid analyser (H835-50, Hitachi, Tokyo, Japan), and the amount of free amino acids was measured by HPLC. After HPLC purity and MALDI-TOF-MS analysis, we found that the content with a relative molecular mass greater than 2000 was 1.96%, between 1000 and 2000 was 5.27%, and less than 1000 (small molecule oligopeptides) in BCOP was 92.77%; the content of free amino acids amounted to 2.73%. The amino acid composition is shown in Table 5.

**Table 5.** Amino acid composition of BCOP.

**Amino acid**	**Amino acid composition of BCOP (g/100 g)**
Asp	0.02
Glu	0.12
Ser	0.00
His	0.01
Gly	0.05
Thr	0.09
Arg	1.12
Ala	0.09
Tyr	0.28
Cys	0.01
Val	0.08
Met	0.07
Phe	0.22
Ile	0.06
Leu	0.35
Lys	0.15
Pro	0.01

Abbreviation: BCOP: bovine bone collagen oligopeptides.

AIN-93G rodent diet were purchased from HFK Bioscience Co. Ltd. (Beijing, China). The dietary ingredients were thoroughly mixed together into granules and then air-dried at room temperature.

### Animals and experimental procedure

A total of 168 ICR male mice (6–8 weeks old, 18–22 g) were obtained from Animal Service of Health Science Center, Peking University, Beijing, China. Mice were housed two per plastic cages at controlled temperature (21–25°C), relative air humidity (50 ± 5%), and 12 h:12 h light/dark cycles (light on 07:00–19:00), with free access to chow and water. All animals were handled according to the guidelines of the Principle of Laboratory Animal Care (NIH publication No. 85–23, revised 1985) of the Peking University Animal Research Committee (https://www.lab.pku.edu.cn/).

Before starting the experiment, the animals were acclimatized to laboratory environment for one week. Two wound models (the full-thickness incision and full-thickness excision) were included in this study. Mice were randomly divided into two wound model groups (*n* = 96 in full-thickness excision model, and *n* = 72 in full-thickness incision model). Within each wound model group, mice were then randomly divided into four dose groups (the model control group, low-dose BCOP group, medium-dose BCOP group, and high-dose BCOP group, namely NC, BCOP-L, BCOP-M and BCOP-H). Each dose group consisted of 24 and 18 animals in full-thickness excision and full-thickness incision group, respectively. The study dose of BCOP used in mice was converted from a human dose of 10 g/kg. Mice in BCOP-treated groups were intragastrically administered BCOP (0.75 g/kg, 1.50 g/kg and 3.0 g/kg), while NC were administered distilled water as vehicle. Oral administration began on the day of wounding and was maintained daily until sacrifice.

### Surgical intervention

#### Full-thickness excision wound model

After shaving the dorsum, two 6-mm diameter full-thickness excision wounds were performed under aseptic conditions on the left and right side of each mouse’s back after being intraperitoneally anaesthetised with pentobarbital sodium. The wounding day was considered as day zero. Six mice in each dose group were randomly selected for the detection of indexes on days 4, 8, 12 and 16, respectively. Blood samples were obtained from mice’s eyeballs and then sacrificed. The serum was separated for biochemical assays by centrifugation (3500 × g for 10 min at 4°C). The wound tissue was cut out at a distance of 3 mm from the edge of the wound and used as experimental samples. Samples from the left wounds were used for measuring hydroxyproline levels, and the right samples were used for the determination of tensile strength and histology.

#### Full-thickness incision wound model

After being intraperitoneally anaesthetised with pentobarbital sodium, two 2.0-cm long, parallel, full-thickness incision wounds were made on the left and right side of each mouse’s shaved dorsum under aseptic conditions. Whereafter, both incisions were stitched tightly and neatly using a No. 9 curved needle and No. 000 surgical thread at 0.5 cm intervals. The wounding day was considered as day zero. Six mice in each dose group were randomly selected for the detection of indexes on days 3, 7 and 11, respectively. Remove sutures on day 7. Blood samples were obtained from mice's eyeballs and then sacrificed. The serum was separated for biochemical assays by centrifugation (3500 × g for 10 min at 4°C). The wound tissue was cut out at a distance of 3 mm from the edge of the wound and used as experimental samples. Samples from the left wounds were used for measuring hydroxyproline levels, and the right samples were used for the determination of tensile strength and histology.

### Evaluation of wound healing related parameters

#### Measurement of the size of wound un-closure

In full-thickness excision wound model, the size of wound un-closure was measured by tracing the wound margin using a transparent paper every 3 days. The size was measured from day 1.

#### Measurement of tensile strength

Tensile strength, the ability to resist fracture under tension, indicates the extent to which the repaired tissue resists damage under tension, and may indicate the quality of the repaired tissue. In full-thickness excision wound model, the wound tissues on day 7 and 11 were prepared into skin islands of 0.5 cm × 1.0 cm, and the incisions were placed on the device connected to JZ300 force transducer working with a Biomedical Signal Acquiring Processing Systems (Beijing MicroStar Technology Development Co., Ltd., Beijing, China). Manually stretch the tissue through a vernier mechanism. Note the fracture strength as the wound cracks. After measuring the thickness of the wound tissue by a micrometer, the ratio of the breaking strength to the surface area of the wound (mm^2^) was recorded as the tensile strength.

### Biochemical assay

The levels of albumin (ALB), prealbumin (PA) and transferrin (TRF) in serum were determined by Olympus AU400 automatic biochemistry analyzer (Olympus, Tokyo, Japan). The level of hydroxyproline (Hyp) in wound tissue was detected according to the kit’s instructions. The levels of interleukin (IL)-8, IL-10, tumor necrosis factor (TNF)-α, chemokine (C-C motif) ligand 2 (CCL2), C-reactive protein (CRP) and SDF-1α in mice serum were measured by an enzyme linked immunosorbent assay (ELISA). All detection kits were purchased from Beyotime Institute of Biotechnology (Beijing, China).

### Histological observation

Remove the entire wound (including a 5 mm margin of unwounded skin) to the fascia on on day 3, 7 and 11 in full-thickness incision wound model, as well as day 8 and 16 in full-thickness excision wound model. The samples were fixed in 10% formalin buffer for 24 hours and then subjected to conventional paraffin embedding treatment. 5 μm-thick sections were mounted on glass slides, dewaxed, rehydrated to distilled water, and stained with hematoxylin and eosin (H & E) or Masson's Trichrome. Two pathologists magnified the magnification of an Olympus IX70 inverted microscope (Olympus, Tokyo, Japan) from 40 to 200 times without understanding the previous treatment.

### Quantitative real-time PCR and analyses of VEGF mRNA

The entire wound tissue was immediately separated on day 11 in full-thickness incision wound model, as well as day 16 in full-thickness excision wound model. And total RNA was extracted from wound tissue using Ribospin (GeneAll, Inc., Seoul, Korea). The ABI 7300 real-time PCR detection system was used for real-time reverse transcription-PCR to detect target genes’ RNA expression. Use the M-MLV kit (Invitrogen) to determine reverse transcription-polymerase chain reaction (RT-PCR) analysis of target mRNA levels. The special primers were as follows: vascular endothelial growth factor (VEGF), forward 5′-ACG AAGTGGTGAAGTTCATGGATG-3′ and reverse 5′-TTC TGTATCAGTCTTTCCTGGTGAG-3′; GAPD, forward 5′-GCCAAAGGGTCATCATCTC-3′ and reverse 5′-GTAGAGGCAGGGATGATGTT-3′. After normalizing the target mRNA value to the GAPDH mRNA level, the target mRNA value was measured by comparison with the control sample, then the comparison period threshold (^ΔΔ^Ct) method is used for calculation.

### Statistical analysis

Statistical analyses were performed using SPSS software (version 17.0, SPSS Inc., Chicago, IL, USA). All data were expressed as mean ± standard deviation (SD). The data of each group were statistically analyzed by one-way analysis of variance. All reported *p*-values were two-sided. *p* < 0.05 was considered significant.
